# ENPP1-Fc prevents mortality and vascular calcifications in rodent model of generalized arterial calcification of infancy

**DOI:** 10.1038/ncomms10006

**Published:** 2015-12-01

**Authors:** Ronald A. Albright, Paul Stabach, Wenxiang Cao, Dillon Kavanagh, Isabelle Mullen, Alexander A. Braddock, Mariel S. Covo, Martin Tehan, Guangxiao Yang, Zhiliang Cheng, Keith Bouchard, Zhao-Xue Yu, Stephanie Thorn, Xiangning Wang, Ewa J. Folta-Stogniew, Alejandro Negrete, Albert J. Sinusas, Joseph Shiloach, George Zubal, Joseph A. Madri, Enrique M. De La Cruz, Demetrios T. Braddock

**Affiliations:** 1 Department of Pathology, Yale University School of Medicine, New Haven, Connecticut 06510, USA; 2 Department of Molecular Biophysics and Biochemistry, Yale University, New Haven, Connecticut 06520, USA; 3 Alexion, Cheshire, Connecticut 06410, USA; 4 Department of Medicine and Diagnostic Radiology, Yale University School of Medicine, New Haven, Connecticut 06510, USA; 5 WM Keck Biotechnology Research Laboratory, Yale University School of Medicine, New Haven, Connecticut 06511, USA; 6 Biotechnology Core Laboratory, National Institute of Diabetes and Digestive Kidney Diseases, National Institutes of Health, Rockville, Maryland 20852, USA; 7 Z-Concepts LLC, East Haven, Connecticut 06512, USA

## Abstract

Diseases of ectopic calcification of the vascular wall range from lethal orphan diseases such as generalized arterial calcification of infancy (GACI), to common diseases such as hardening of the arteries associated with aging and calciphylaxis of chronic kidney disease (CKD). GACI is a lethal orphan disease in which infants calcify the internal elastic lamina of their medium and large arteries and expire of cardiac failure as neonates, while calciphylaxis of CKD is a ubiquitous vascular calcification in patients with renal failure. Both disorders are characterized by vascular Mönckeburg's sclerosis accompanied by decreased concentrations of plasma inorganic pyrophosphate (PP_i_). Here we demonstrate that subcutaneous administration of an ENPP1-Fc fusion protein prevents the mortality, vascular calcifications and sequela of disease in animal models of GACI, and is accompanied by a complete clinical and biomarker response. Our findings have implications for the treatment of rare and common diseases of ectopic vascular calcification.

## Abstract (web summary)

Generalized arterial calcification of infancy (GACI) is a terminal disease caused by the ENPP1 enzyme deficiency. Here, Albrigh *et al*. show that ENPP1 enzyme replacement therapy prevents the ectopic calcifications and mortality in mice with GACI, suggesting a novel treatment for vascular calcification in humans.

## Graphical abstract

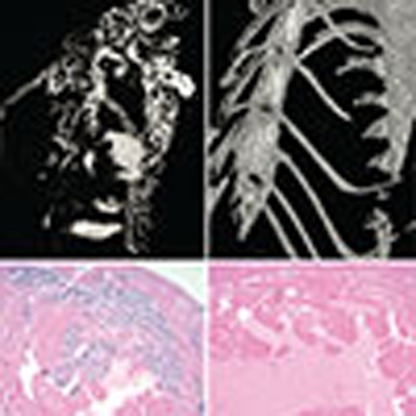

Generalized arterial calcification of infancy (GACI) is an ultra-rare neonatal disease characterized by infantile onset of widespread arterial calcifications in large and medium-sized vessels resulting in cardiovascular collapse and death in the neonatal period. The disease presents clinically with heart failure, respiratory distress, hypertension, cyanosis and cardiomegaly. The prognosis is grave, with older reports of an 85% mortality rate at 6 months^1^, while recently intensive treatment with bisphosphonates has lowered mortality to 55% at 6 months^2^. Tempering this apparent progress is the severe skeletal toxicity associated with prolonged use of etridonate in patients with GACI (ref. 3), the observation that the limited available data makes it difficult to determine if bisphosphonate treatment is truly protective or reflects the natural history of the disease in less effected patients, and the ineffectiveness of bisphosphonates to prevent mortality in some patients even when instituted early^4^,^5^,^6^,^7^.

The overall incidence of GACI is rare, with 200 reported cases in the medical literature^8^ and a disease frequency of one in 391,000 (ref. 9). Although the disease was first described by Bryant and White in 1901 (ref. 10), it was not until 2000 that Rutsch *et al*.^11^ noted that serum plasma inorganic pyrophosphate (PP_i_) levels and ectonucleotide pyrophosphatase/phosphodiesterase 1 (ENPP1) enzymatic activity were significantly impaired in GACI patients. ENPP1 (also known as PC-1) is the founding member of the ENPP or NPP family of enzymes, which are characterized by phosphodiesterase activity^12^, and is a type II extracellular membrane bound glycoprotein located on the mineral-depositing matrix vesicles of osteoblasts and chondrocytes, as well as the vascular surface of cerebral capillaries^13^. ENPP1 is the primary source of extracellular PP_i_ in the body, and hydrolyzes extracellular ATP into AMP and PP_i_ (refs 12, 14). PP_i_ acts as a potent inhibitor of mineralization, presumably by occupying some of the P_i_ sites on the surface of nascent or growing hydroxyapatite crystals, thereby creating irregularities that slow or terminate crystal growth^14^,^15^. Rutsch *et al*. discovered that inactivating mutations in ENPP1 account for 75% of GACI patients^2^,^11^,^16^ and have contributed to recent observations that a sizable fraction of the remaining patients result from inactivating mutations in the ATP dependent membrane transporter ABCC6 (refs 17, 18, 19, 20, 21, 22). ABCC6 mutations result in decreased extracellular concentrations of nucleoside triphosphates through an unknown mechanism^23^, thereby limiting ENPP1's metabolism of ATP into extracellular PP_i_. The identification of both ENPP1 and ABCC6 inactivating mutations as genetic etiologies for GACI therefore links the same biochemical pathway to unregulated tissue mineralization in both genetic variants of GACI.

Despite the multiple genetic etiologies and multifactorial nature of the expression, progression, and severity of medial wall vascular calcification, we hypothesized that disruption of ENPP1's extracellular purinergic metabolism accounts for the pathologic sequela and mortality associated with GACI and that enzyme replacement therapy with ENPP1 is a tractable therapeutic approach. To test this hypothesis, we employed ENPP1-asj mice (Jackson Laboratory), which carry an inactivating mutation in *enpp1* such that homozygotes (*enpp1*^*asj/asj*^) have markedly reduced ENPP1 function. To enhance the disease phenotype, the mice were maintained on the an ‘acceleration diet' *in utero*^24^,^25^. Here we demonstrate that daily subcutaneous doses of a biologic agent comprising the extracellular domain of ENPP1 fused to the Fc region of human IgG1 eliminates the vascular calcifications, mortality, myocardial infarctions, and sequela of GACI in *enpp1*^*asj/asj*^ mice, and that this clinical response is accompanied by normalization of serum and tissue PP_i_ concentrations.

## Results

### Natural history study of GACI in *enpp1*^*asj/asj*^ mice

**Figure 1:**
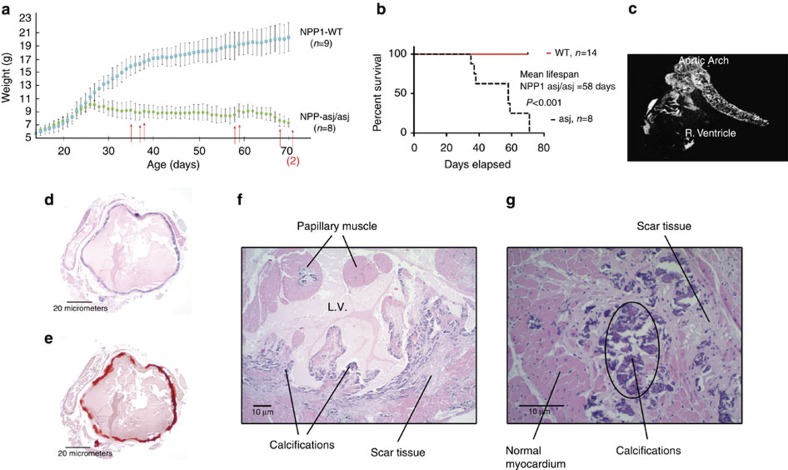
Natural history study. (**a**) Daily weights of *enpp1*^*wt*^ (cyan squares, *n*=9 animals) and *enpp1*^*asj/asj*^ mice (green circles, *n*=8 animals) on the acceleration diet *in utero* over a 70 day period. Mean average weights are plotted with s.d's denoted by error bars. A failure to thrive point is noted in the *enpp1*^*asj/asj*^ cohort at day 26, when the weights diverge from *enpp1*^*wt*^. Death events are marked with red arrows. (**b**) Mean survival of *enpp1*^*asj/asj*^ was 58 days. No deaths were observed in the *enpp1*^*wt*^ cohort. Analysis by log-rank (Mantle–Cox) test yields a *χ*^2^ of 15.73 and *P* value of <0.0001. (**c**) *enpp1*^*asj/asj*^ animals displayed dramatic calcifications in heart and aorta visible on micro-CT scans. (**d**,**e**) Histology of *enpp1*^*asj/asj*^ mice, aorta (Hematoxylin and Eosin (H&E) and Alizarin red). Aortas of *enpp1*^*asj/asj*^ mouse all possessed near circumferential calcifications that were pervasive in the vascular walls, as illustrated by Alizarin red staining of the aortas. Scale bar, 20 μm (**f**). Histology of *enpp1^asj/asj^* mice, left ventricle. Extensive calcifications surrounded by scar tissue revealing the presence of repeated, old and healed myocardial infarctions. Scale bar, 10 μm (**g**). Histology of *enpp1*^*asj/asj*^ Mice, Septum (H&E). More typically, the *enpp1*^*asj/asj*^ mice displayed small foci of calcifications with surrounding scar tissue as seen here in the myocardial septum, also diagnostic of previous myocardial infarctions. Scale bar, 10 μm.

When fed an acceleration diet, the daily weights of *enpp1*^*asj/asj*^ mice diverged from wild-type (WT) siblings pairs at day 26, when the ENPP1-asj/asj mice experienced a ‘failure to thrive' event and began to lose weight (Fig. 1a). After day 26 the *enpp1*^*asj/asj*^ animals displayed progressive stiffness and reductions in physical activity. All of the *enpp1*^*asj/asj*^ animals died between days 35–71, with a median lifespan of 58 days (Fig. 1b). The presence of calcifications in *enpp1*^*asj/asj*^ and *enpp1*^*wtj*^ mice was evaluated via post-mortem by micro-computed tomography (CT) scans (Fig. 1c) and histologic sections taken from the heart, aorta and kidneys (Fig. 1d–g). Approximately one-third of the *enpp1*^*asj/asj*^ mice had visible calcifications in their hearts, and two-thirds had calcifications in their aortas visible by micro-CT imaging (Table 1). These percentages increased to 100% on histologic examination, which also showed that many of the animals had dramatic nearly circumferential calcifications in their aortic walls (Fig. 1d,e). Histologic examination also revealed that 100% of the coronary arteries possessed arterial wall calcifications and that 70% of the animals had focal or confluent areas of myocardial necrosis consistent with myocardial infarction (Fig. 1f,g). Conversely, the *enpp1*^*wt*^ mice displayed none of these abnormalities. These findings demonstrate that the animal model recapitulates GACI in humans, which is characterized by prominent calcifications of the large and medium sized arteries and a cardiac demise.

### Design and characterization of therapeutic

**Figure 2:**
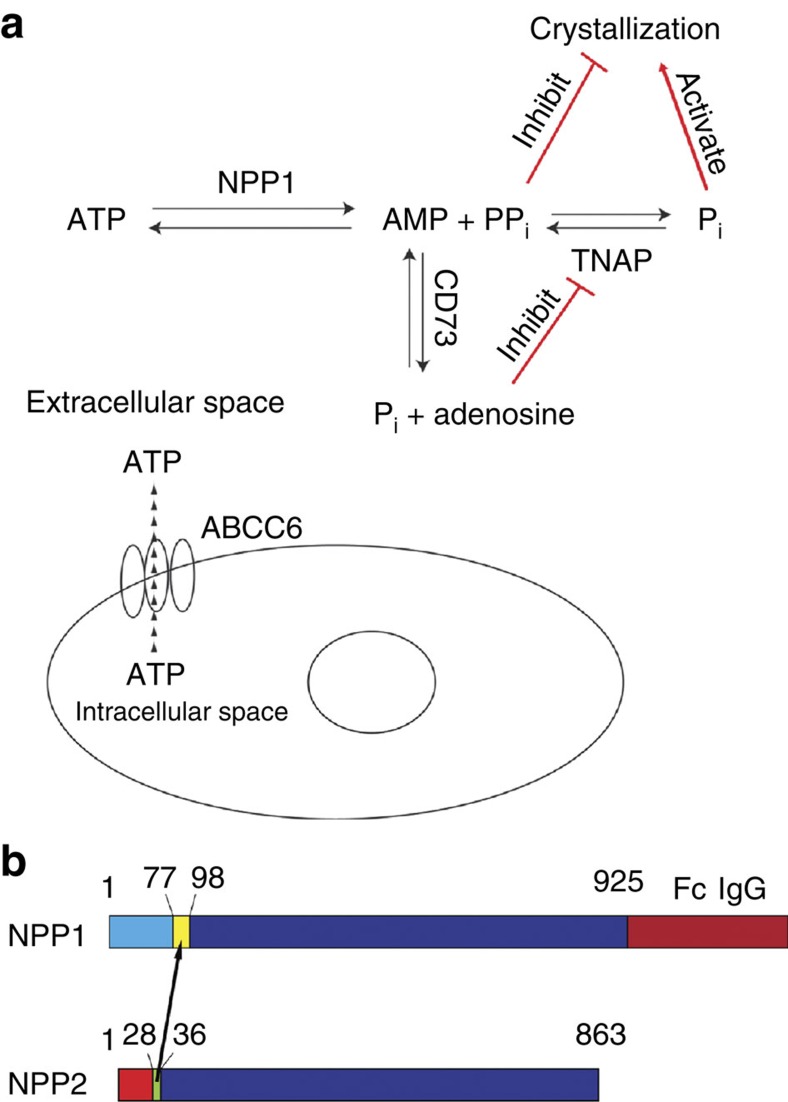
Metabolic pathways and therapeutic design. (**a**) Metabolic pathways of interest. ENPP1 converts extracellular ATP into AMP and PP_i_, TNAP converts PP_i_ into P_i_, and CD73 converts AMP into adenosine and P_i_. ABCC6 is a membrane transporter that increases the extracellular concentration of NTP via an indirect unknown mechanism (shown as a dashed line). Loss of function mutations in TNAP result in familial hypophosphatasia. Loss of function mutations in ENPP1 result in GACI, loss of function mutations in ABCC6 result in pseudoxanthomatous elasticum, and loss of function mutations in CD73 results in a disease of arterial and joint calcification termed ‘ACDC'. TNAP, tissue-nonspecific alkaline phosphatase. (**b**) Design of ENPP1 protein therapeutic. To produce a soluble recombinant protein, a segment of the extracellular region of NPP2 containing a furin cleavage site was substituted into ENPP1 (ref. 26) and the Fc portion of IgG1 was appended to the c-terminus.

**Figure 3:**
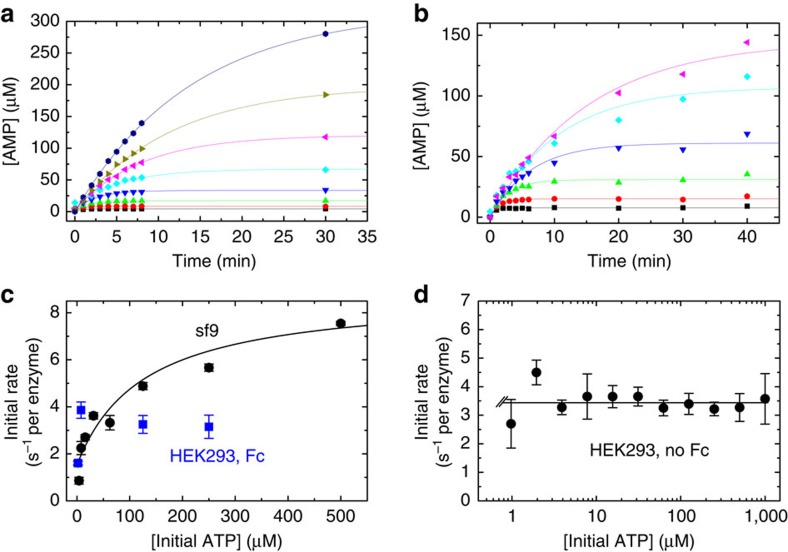
Steady state kinetics of hENPP1-Fc. Time courses of AMP formation measured by HPLC analysis after addition of (**a**) 50 nM hNPP1 purified from sf9 cells to (from bottom to top) 3.9 (black), 7.8 (red), 15.6 (green), 31.3 (blue), 62.5 (cyan), 125 (magenta), 250 (dark yellow) and 500 (navy) μM ATP or (**b**) 10 nM hNPP1 from HEK293 cells to (from bottom to top) 7.8 (black), 15.6 (red), 31.3 (green), 62.5 (blue), 125 (cyan) and 250 (magenta) μM ATP. The smooth curves though the data are fits obtained by non-linear kinetic time course analysis^39^. (**c**) Comparison of ENPP1 expressed in insect cells with ENPP1-Fc expressed in mammalian cells. NPP1 (sf9, black circles) is human ENPP1 produced in baculovirus-infected cells while NPP1 (HEK293, Fc and blue squares) is human ENPP1-Fc produced in HEK293 cells. The initial rate of ATP cleavage by ENPP1-Fc from HEK293 cells is essentially the same at [ATP] greater than 7.8 μM, yielding a *k*_cat_ (the average of the rates ⩾7.8 μM) of 3.4 (±0.4) s^−1^ per enzyme. The initial rate at 2.0 μM ATP concentration is about a half of the *k*_cat_ value. We therefore estimate a *K*_M_ ∼ 2 μM for ATP hydrolysis by hNPP1-Fc protein. (**d**) ATP concentration dependence of the initial hydrolysis rate of ENPP1 (no Fc) purified from HEK293 cells. The reaction displays a *k*_cat_ of 3.46 (±0.44) s^−1^ per enzyme, estimated from the average of all ATP concentrations ⩾2 μM. At 1 μM initial ATP (the first data point), the hydrolysis rate is slightly less than *k*_cat_ values. We therefore estimate a *K*_M_ <2 μM for ATP hydrolysis by ENPP1 (no Fc) purified from HEK293 cells.

To produce soluble, recombinant ENPP1 for *in vivo* use, we modified our previous expression construct^26^ by fusing ENPP1 to the Fc domain of IgG1 (hereafter referred to as hENPP1-Fc and mENPP1-Fc for the human and mouse protein isoforms, respectively, Fig. 2b) and expressed the fusion protein in stable mammalian (HEK293) cell lines (Supplementary Note 1). The effect of expressing the hENPP1-Fc in mammalian, as compared to our previous studies in insect cells^26^, altered the Michaelis–Menton kinetics by reducing (tightening) the Michaelis constant *K*_M_ for ATP substrate by over two orders of magnitude, while also reducing (slowing down) the *k*_cat_ by a factor of 2–3 (Fig. 3a–c). The altered Michaelis–Menton constants could reflect either the change in expression system from baculovirus-infected insect cells to mammalian cells or the addition of the Fc tag. To differentiate between these two possibilities, we expressed hENPP1 without an Fc tag in mammalian (HEK293) cell lines to directly compare the kinetics of ENPP1 with and without an Fc tag. We found that the kinetic constants of the hNPP1-Fc and hNPP1 enzymes expressed in mammalian cells were essentially identical (Fig. 3d), suggesting that the alteration in kinetic constants was due to the change in expression systems.

**Figure 4:**
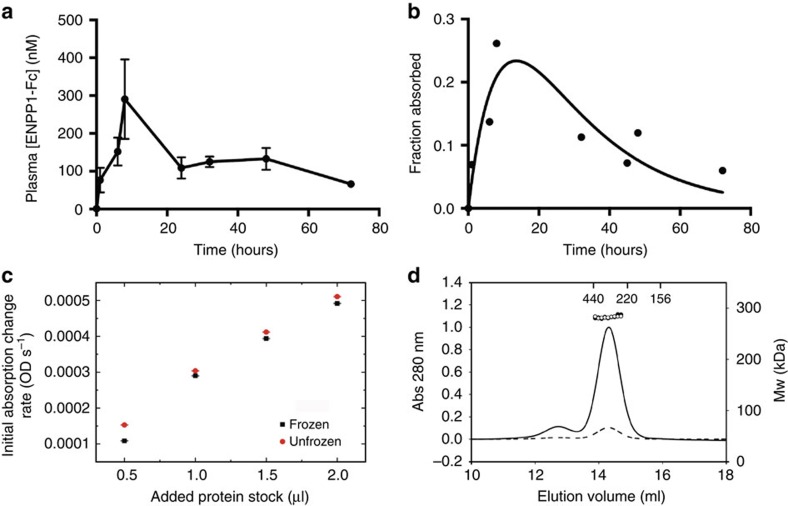
Characterization of ENPP1-Fc. (**a**) Pharmacokinetics of absorption and excretion. 15 C57B6 mice were injected with 10 mg kg^−1^ ENPP1-Fc and blood was collected at the indicated times. The concentration of ENPP1-Fc was estimated from the activity units of enzyme as measured by TMP assay and each data point represents measurement in 5 animals except for time=0, which represents 15 animals. The mean value is plotted, and error bars represent s.d. in the measurements. A *C*_max_ of ≈300 nM is reached at 8 h, and the area under the curve is calculated as 9 μM h^−1^. (**b**) To derive the pharmacokinetic constants of drug absorption and elimination we plotted the fraction of dosed drug absorbed in the plasma from a single 10 mg kg^−1^ subcutaneous dose over time and fit the resulting curve with the equation 
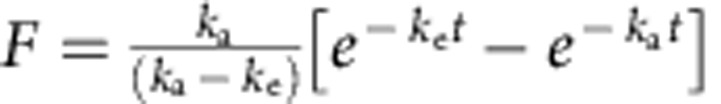
 to obtain the elimination and absorption constants *k*_e_ and *k*_a_, respectively. The resulting curve yielded values for *k*_e_=0.107±0.016 h^−1^ and *k*_a_=0.048±0.008 h^−1^. The goodness of the fit yielded an *R*^2^=0.7573 and an absolute sum of squares=0.01. (**c**) Stability of ENPP1 therapeutic. ENPP1-Fc Ap3A activity was seen to be stable to freeze-thaw cycle in PBS following storage at −80 °C. (**d**) Molecular weight of ENPP1-Fc determined by SEC-MALLS/RI/UV. Weight average molar masses (Mw) are plotted for analyses of ENPP1-Fc protein at various concentrations. Lines correspond to UV traces of the protein eluting from the SEC column monitored at 280 nm (left axis). Molar masses were recorded every sec across the elution profile (circles; right axis); for clarity only every 20th measurement of molar mass is plotted. ENPP1-Fc protein was analysed in a concentration range from 100 nM (dashed line, empty circles) to 1 μM (solid line, filled circles) measured at the apex of the eluting peak. The elution's position of protein standards used for validation of SEC-MALLS/UV/RI performance are marked. The SEC-MALLS/UV/RI analysis yielded MW of 274±38 KDa with a glycosylation estimate of 0.05 g of sugars per gram of polypeptide.

To understand the pharmacokinetics of ENPP1-Fc we determined plasma concentrations of drug following a single subcutaneous injection over time. Fifteen mice were administered a single subcutaneous dose of 10 mg kg^−1^ and blood was collected from tail veins to quantitate ENPP1-Fc in the plasma. The concentration ENPP1-Fc reaches a maximal plasma concentration of ∼300 nM (*C*_max_) within 8 h (*t*_max_) and remains elevated at 100 nM for 72 h after dosing (Fig. 4a). The integral of the plotted concentration–time curve, or area under the curve, is 9 μM h^−1^. Plotting the data as fraction of drug absorbed over time allows for the determination of the elimination (*k*_e_) and absorption (*k*_a_) constants by fitting the data to the equation for the total systemic absorption of a drug administered at a subcutaneous depot at time zero,


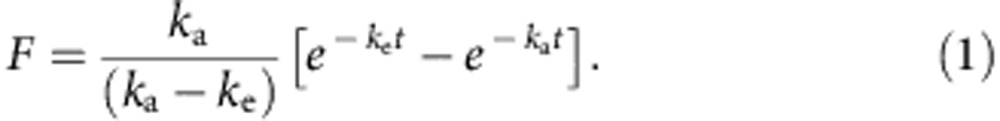


This analysis yield a *k*_e_=0.107±0.016 h^−1^ and a *k*_a_=0.048±0.008 h^−1^, which yields an elimination half life (*t*_1/2_)=6.5 h (Fig. 4b).

The activity of ENPP1-Fc was noted to diminish over a 30 day period when stored at 4 °C, but the enzyme could be frozen at −80 °C and retain nearly complete activity on thawing (Fig. 4c). The enzyme was therefore stored as a frozen stock solution after purification until needed. The oligomerization state of ENPP1-Fc at 1 μM and 100 nM was determined by size exclusion chromatography coupled with light scattering, refractive index, and absorbance (ultraviolet) detection (SEC-MALLS/RI/UV)^27^, which allows for a determination of the molecular weight (MW) of glycosylated proteins from the relationship of molecular weight and the ratio of laser light scattering, ultraviolet and refractive index (RI) signals in the absence of knowledge of the extend of glycosylation^28^. The results of the SEC-MALLS analysis revealed that the MW of the ENPP1-Fc protein represents a dimer over the concentration range of 100 nM–1 μM, with an experimentally determined MW of 274±38 KDa (Fig. 4d). This agrees well with the calculated MW of 252 KDa for an ENPP1-Fc dimer. The experimental approach also allows for an independent estimation of the glycosylation level of the fusion protein, which was determined to be ∼5% according to the method of Hayashi *et al*.^29^

Following purification, ENPP1-Fc was dialysed into PBS supplemented with Zn and Ca^2+^ (PBS_plus_) concentrated to between 5 and 7 mg ml^−1^ and frozen at −80 °C in aliquots of 200–500 μl. Aliquots were thawed immediately before use, and the specific activity of the solution was adjusted to 31.25 a.u. ml^−1^ (or ≈0.7 mg ml^−1^ depending on the preparation) by dilution in PBS_plus_.

### Therapeutic proof of concept in *enpp1*^*asj/asj*^ mice

Dosing was performed according to activity units (a.u.) per kg animal weight to account for variations in specific activity in different protein preparations. The specific activity of the enzyme varied with each protein preparation, and because the clinical response was noted to be highly dependent on enzyme specific activity, we rejected protein preparations with specific activities of <40 a.u. mg^−1^. To establish initial dosing levels for the proof of concept study we performed dose escalation trials in limited numbers of animals (1–2 per dose level). While both the human and mouse version ENPP1 were used in the dose escalation trials, the proof of concept study was performed with the mouse isoform of ENPP1-Fc (mENPP1-Fc). *enpp1*^*asj/asj*^ mice were dosed daily on the fourteenth day of life with subcutaneous injections of mENPP1-Fc and weekly with intra-peritoneal injections of GK 1.5, the latter added to minimize immune rejection of recombinant protein^30^. Subcutaneous doses of mEnpp1-Fc at 500 a.u. kg^−1^ qD demonstrated a strong early response in weight with an absence of the observed ‘failure to thrive' crisis observed in undosed *enpp1*^*asj/asj*^ animals (Supplementary Fig. 1). This dose corresponded to between 6–10 mg kg^−1^ of ENPP1-Fc, depending on the specific activity of the protein preparation.

**Figure 5:**
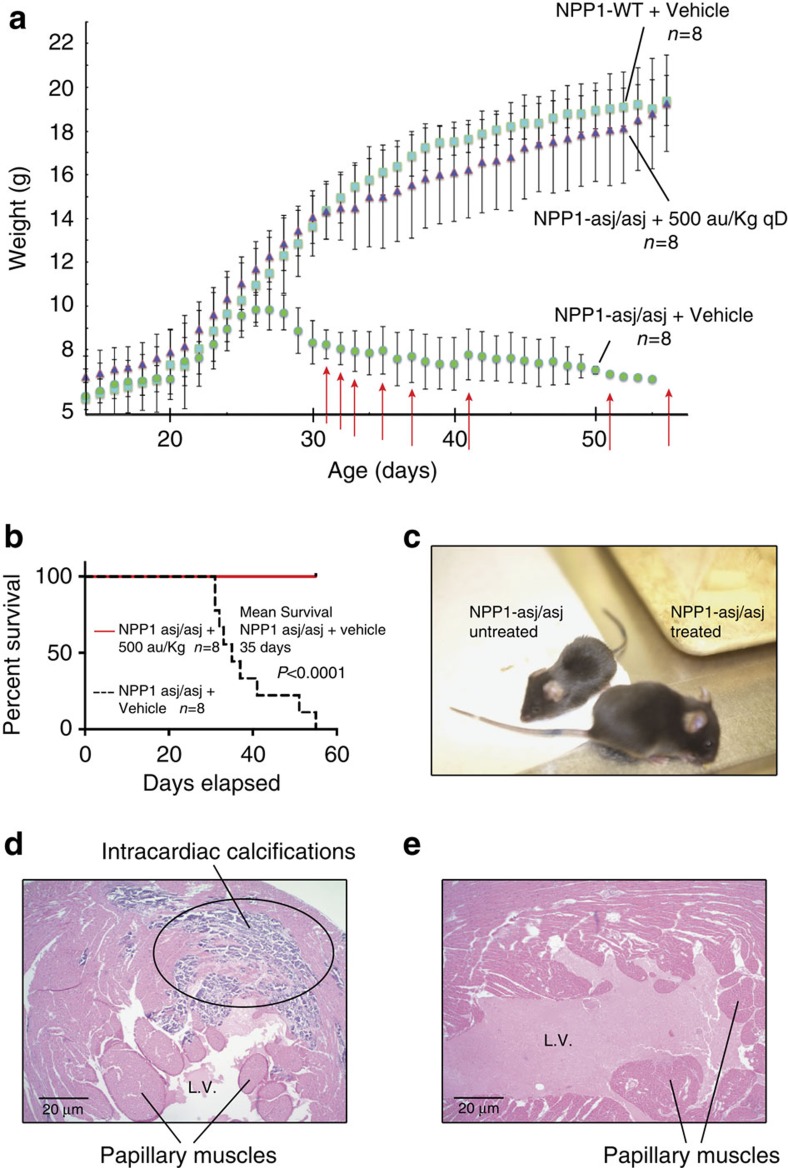
Proof of Concept Study. (**a**) Daily animal weights. The mean daily weights of *enpp1*^*wt*^ (cyan squares, *n*=8), treated *enpp1*^*asj/asj*^ (purple triangles, *n*=8), and untreated *enpp1*^*asj/asj*^ mice (green circles, *n*=8) are plotted with s.d.'s denoted by error bars. Dosing with eNPP1-Fc and weighing commenced on day 14. Treatment consisted of daily injections of 10 mg kg^−1^ ENPP1-Fc formulated in PBS_plus_ and weekly injections of Gk 1.5. Untreated *enpp1*^*asj/asj*^ and *enpp1*^*wt*^ received daily injections of PBS_plus_ and weekly injections of GK 1.5. Deaths in the untreated *enpp1*^*asj/asj*^ cohort are denoted by red arrows on the day of death. No deaths were noted in the *enpp1*^*wt*^ or the treated *enpp1*^*asj/asj*^ cohort. (**b**) Survival curves, proof of concept study. The survival of treated and untreated *enpp1*^*asj/asj*^ animals are plotted as a solid red line and dashed black line, respectively. Analysis by Log-rank (Mantle–Cox) test yields a *χ*^2^ of 13.18 and *P* value of 0.003. (**c**) Phenotypic comparison, treated and untreated *enpp1*^*asj/asj*^ mice. There is a dramatic size difference in the treated and untreated animals, and a marked difference in the mobility and health of the animals, best seen in Supplementary Movie 1. (**d**) Left ventricle histology, untreated asj/asj mouse Hematoxylin and Eosin (H&E),displaying large focus of calcifications and micro-infarctions in the free wall. Scale bar, 20 μm. (**e**) Left ventricle histology, treated *enpp1*^*asj/asj*^ mice (H&E). None of the treated *enpp1*^*asj/asj*^ mice displayed abnormal L. ventricular histology. Scale bar, 20 μm.

On the basis of the results of the dose escalation trials we chose to enrol a cohort of 8 *enpp1*^*asj/asj*^ animals dosed with mENPP1-Fc at 500 a.u. kg qD and weekly intra-peritoneal injections with GK 1.5 (Fig. 5). We included a control groups (*enpp1*^*wt*^ and *enpp1*^*asj/asj*^) dosed daily with vehicle and weekly with GK 1.5 in an identical manner as the dosed cohort, and the study duration was shortened to 55 days. All 8 treated *enpp1*^*asj/asj*^ animals survived the full 55 days of the trial, with a dramatic clinical response observed in treated animals (Supplementary Movie 1), while the median lifespan of the untreated *enpp1*^*asj/asj*^ animals decreased from 58 to 35 days in the therapeutic trial, perhaps resulting from the weekly intra-peritoneal injections of the GK 1.5 immunosuppressant. The untreated *enpp1*^*asj/asj*^ animals also all experienced a failure to thrive crisis at day 26, followed by weight loss and mobility restriction progressing variably to paralysis and death over the next 30 days. All but one untreated *enpp1*^*asj/asj*^ animal expired over the 55 day trial, while in contrast all treated *enpp1*^*asj/asj*^ mice gained weight comparable to the *enpp1*^*wt*^ mice and displayed no signs of reduced mobility or stiffness (Supplementary Movie 1).

**Figure 6:**
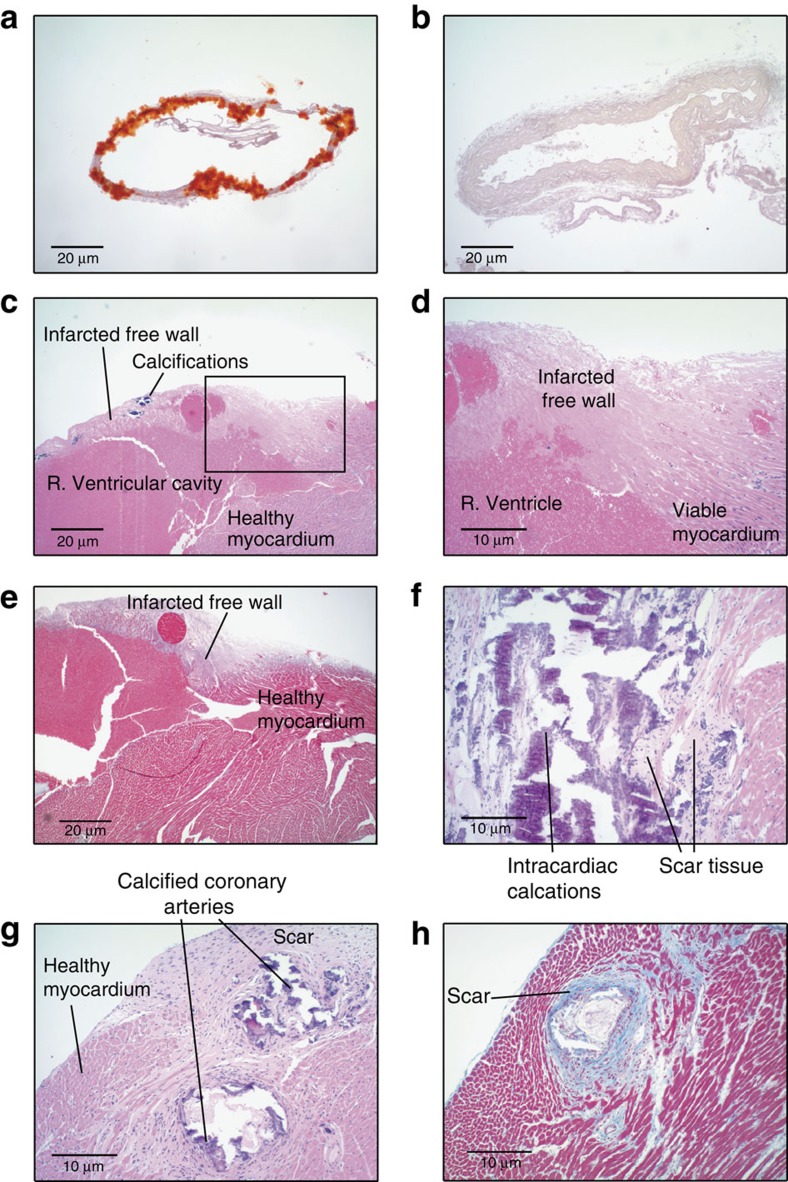
Representative histology, proof of concept study. (**a**,**b**) Aorta (Alizarin red). Untreated *enpp1*^*asj/asj*^ mice displayed nearly circumferential aortic calcifications (**a**), while treated *enpp1*^*asj/asj*^ mice did not (**b**). Scale bar, 20 μm. (**c**) Untreated *enpp1*^*asj/asj*^ mice, right (R) ventricle Hematoxylin and Eosin (H&E). Two untreated *enpp1*^*asj/asj*^ mice had large, confluent, myocardial infarctions in the free wall of the R. ventricle. Scale bar, 20 μm. All treated *enpp1*^*asj/asj*^ mice displayed normal R. ventricle myocardium (not shown). (**d**) Untreated *enpp1*^*asj/asj*^ mice, R. ventricle (H&E) higher power view of the boxed area in panel c. Scale bar, 10 μm. (**e**) Untreated *enpp1*^*asj/asj*^ mice, R. ventricle (Trichrome) Trichrome stains of the R. ventricle in the same animal demonstrates the infarcted free wall. Scale bar, 20 μm. (**f**) Untreated *enpp1*^*asj/asj*^ mice, myocardial septum (H&E). Nearly all animals (77%) displayed intracardiac calcifications surrounded by scar tissue, as demonstrated in this animal in the myocardial septum. Scale bar, 10 μm. (**g**) Untreated *enpp1*^*asj/asj*^ mice, coronary arteries (H&E). All untreated *enpp1*^*asj/asj*^ mice had coronary calcifications, with most displaying circumferential calcifications in coronary arteries surrounded by scar tissue, diagnostic of ischaemia and myocardial infarction. Scale bar, 10 μm. (**h**) Untreated *enpp1*^*asj/asj*^ mice, coronary arteries (Trichrome). Trichrome stains of coronary artery regions of untreated *enpp1*^*asj/asj*^ mice demonstrates increased fibrosis associated with vascular wall calcifications (blue colour and labelled ‘scar'), demonstrating the myocardial injury in the animals. Scale bar, 10 μm.

**Figure 7:**
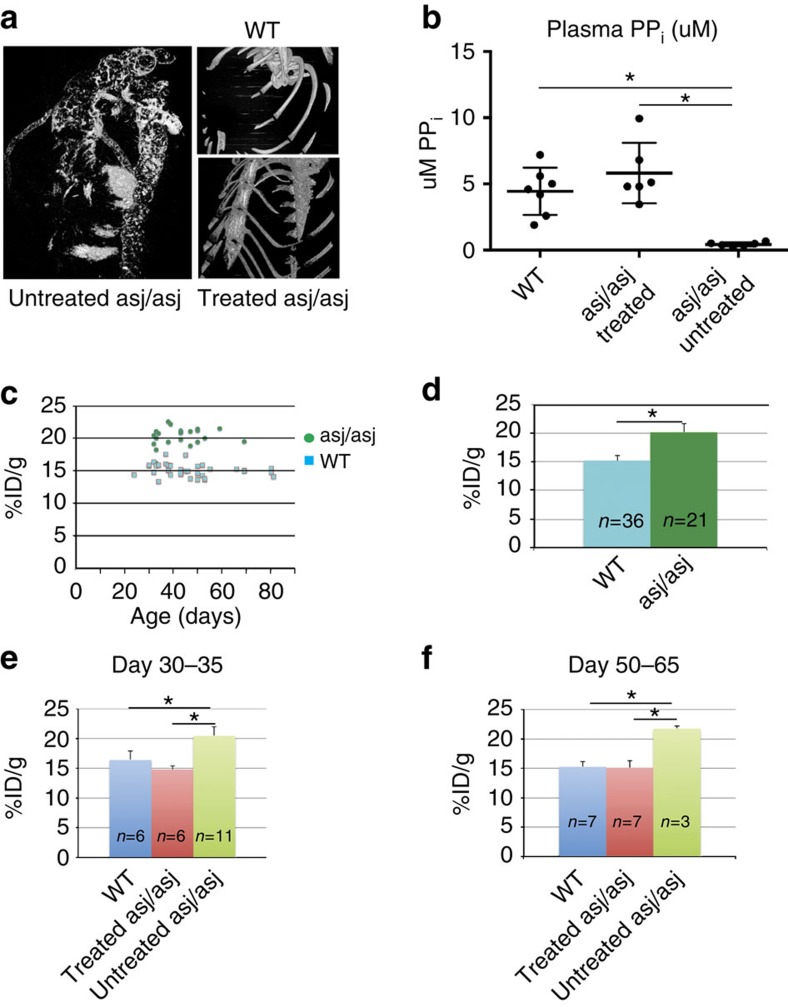
Biomarkers of disease response. (**a**) Postmortem high-resolution micro-CT scans revealed extensive calcifications in untreated *enpp1*^*asj/asj*^ mice in the hearts, coronary arteries, and ascending and descending aortas, but absolutely no calcifications in these organs in the treated *enpp1*^*asj/asj*^ cohort or in *enpp1*^*wt*^ mice. For further imaging, Supplementary Movie 2. (**b**) Plasma [PP_i_] in *enpp1*^*wt*^ and treated and untreated *enpp1*^*asj/asj*^ animals revealed that treatment with mENPP1-Fc increased [PP_i_] in *enpp1*^*asj/asj*^ mice to WT levels, and well above the nearly undetectable levels present in untreated *enpp1*^*asj/asj*^ mice. *P<0.0015, Students two-tailed *t*-test. (**c**,**d**) Per cent uptake of injected ^99m^PYP in heads of WT and asj/asj animals. The per cent uptake of ^99m^PYP in heads of animals in the natural history study were recorded weekly in the WT and asj/asj animals on the acceleration diet, demonstrating that ^99m^PYP uptake remains nearly constant over an 80 day period following birth, but differs markedly between the two experimental groups. (**d**) In the natural history study, the average ^99m^PYP uptake in heads of *enpp1*^*wt*^ animals was around 15% of injected dose over the 80 day period, while the PYP uptake in *enpp1*^*asj/asj*^ animals was around 20% *P<0.001, Students two-tailed *t*-test. (**e**,**f**) ^99m^PYP uptake. The per cent ^99m^PYP uptake in the heads of all experimental groups was recorded in the middle of the study (days 30–35, in **e**) and at the end of the study (days 50–65, in **f**). *enpp1*^*wt*^ and treated *enpp1*^*asj/asj*^ animals had per cent uptake in the skulls around 15%, while the untreated *enpp1*^*asj/asj*^ cohort was at or above 20%. *P<0.001, Students two-tailed *t*-test.

At the conclusion of the study, 100% of the *enpp1*^*asj/asj*^ mice treated with vehicle displayed calcifications in their hearts, aortas and coronary arteries, and 77% of the animals displayed histologic evidence of myocardial infarction (Table 2). In most cases this took the form of small areas of myocardial cell necrosis and single cell drop out in the vicinity of the cardiac calcifications (Figs 5d and 6f–h), but in two animals (22%) there were large, full thickness myocardial infarctions in the free wall of the right ventricle (Fig. 6c–e). Myocardial fibrosis in the myocardial tissue adjacent to regions of coronary artery calcification was a common finding (Fig. 6g,h), illustrating that ischaemia from coronary artery calcification likely accounts for a substantial burden of the myocardial disease. Some untreated *enpp1*^*asj/asj*^ animals displayed dramatic calcifications of coronary arteries, heart, ascending and descending aorta (Supplementary Movie 2). In contrast, none of the *enpp1*^*asj/asj*^ animals treated with ENPP1-Fc displayed cardiac, arterial, or aortic calcification on histology or post-mortem micro-CT (Table 2 and Figs 5e, 6b and 7a). Although renal calcifications are not a feature of human GACI, the *enpp1*^*asj/asj*^ mouse model is noted to have calcifications in the kidneys. Similar to previous findings, we noted light calcifications in the kidneys of ≈60% of the WT animals and 100% of the dosed *enpp1*^*asj/asj*^ mice. The calcifications in these animals were centred in the renal medulla. 100% of the undosed *enpp1*^*asj/asj*^ animals had heavy, extensive calcifications, centred in the outer medulla, with extension into the renal cortex. In comparison to previous reports, the renal calcifications in the dosed animals seem to approximate renal calcifications seen in the heterozygous *enpp*^*WT/asj*^ animals.

### Biomarker response

In addition to survival, daily animal weights, and terminal histology, treatment response was also assessed via post-mortem high-resolution micro-CT scans to image vascular calcifications, plasma PP_i_ concentrations, and ^99m^Tc PP_i_ (^99m^PYP) uptake (Fig. 7a–f and Table 2). The biochemical and physiologic response was complete as measured by all of these parameters. None of the WT or treated *enpp1*^*asj/asj*^ animals were noted to possess any vascular calcifications via micro-CT, in contrast to the dramatic calcifications noted in the aortas, coronary arteries, and hearts of the untreated *enpp1*^*asj/asj*^ cohort (Fig. 7a). In addition, serum PP_i_ concentrations of treated *enpp1*^*asj/asj*^ animals (≈5.2 μM) were elevated to WT levels (4.4 μM) and significantly above untreated *enpp1*^*asj/asj*^ levels (<0.5 μM) (Fig. 7b).

^99m^PYP, an imaging agent typically employed in cardiac imaging and bone remodelling, was used as a marker for treatment response. It is sensitive to areas of unusually high-bone rebuilding activity since it localizes to the surface of hydroxyapatite and then may be taken up by osteoclasts. One would expect increased ^99m^PYP uptake in animals lacking functional ENPP1 since they have reduced plasma [PP_i_] and correspondingly elevated rates of mineralization. To test this hypothesis, we performed weekly *in vivo*^99m^PYP imaging in *enpp1*^*wt*^ and undosed *enpp1*^*asj/asj*^ animals to detect differences in ^99m^PYP uptake between the sibling pairs (Fig. 7c,d). We limited analysis of ^99m^PYP uptake to the head, which is comprised of both enchondral bone (skull) and soft tissue (vibrissae), which are known sites of ectopic calcification in *enpp1*^*asj/asj*^ mice. This also simplified data analysis as the head does not overlap with internal organs showing transient ^99m^PYP uptake (such as the bladder, heart and diaphragm) during the 180° camera rotation that occurs during data collection.

Weekly serial imaging of *enpp1*^*wt*^ and untreated *enpp1*^*asj/asj*^ animals demonstrated that, as expected, uptake of ^99m^PYP in the heads as per cent injected dose was greater in *enpp1*^*asj/asj*^ animals than in *enpp1*^*wt*^ animals, and that changes in ^99m^PYP uptake within experimental groups did not vary significantly over the course of the study (Fig. 7c,d). We therefore chose to measure ^99m^PYP uptake in treated and untreated *enpp1*^*asj/asj*^ animals at two time points—days 30–35 and at the completion of the study (days 50–65). Comparison of these experimental groups demonstrates that ENPP1-Fc treatment returned ^99m^PYP uptake in GACI mice to WT levels (Fig. 7e,f), suggesting that ENPP1-Fc treatment is able to abrogate unregulated tissue, vibrissae and skull mineralization in *enpp1*^*asj/asj*^ mice by raising the extracellular PP_i_ concentrations.

### Reappearance of calcifications following cessation of dosing

**Figure 8:**
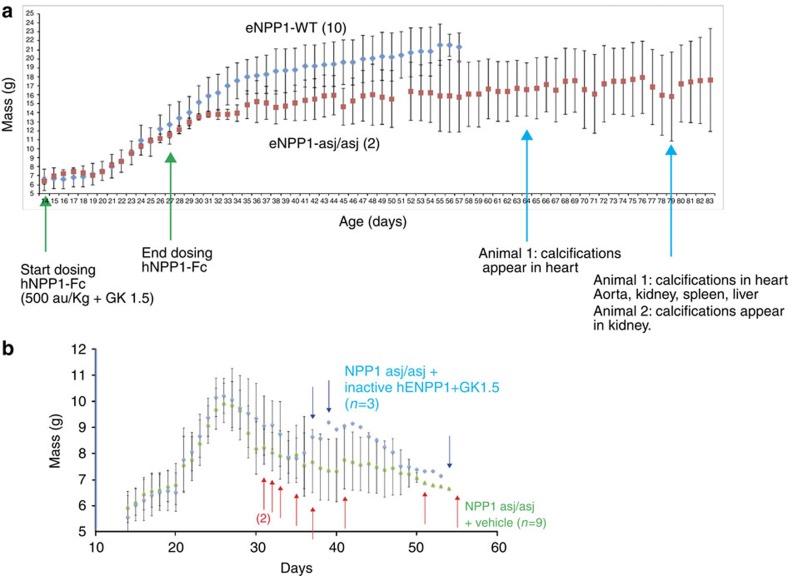
Limited dosing study. (**a**) To determine if calcifications reappear following cessation of dosing, two *enpp1*^*asj/asj*^ animals were dosed daily with hENPP1-Fc between days 14–27 (green arrows), followed by daily dosing with PBS_plus_. Rejection of human version of ENPP1-Fc (hENPP1-Fc) was suppressed during the limited dosing period with weekly doses of GK 1.5. The average daily weights are of *enpp1*^*wt*^ (blue triangles, *n*=10) and *enpp1*^*asj/asj*^ (red squares, *n*=2) mice are plotted with the error bars denoting s.d.'s. The dosed *enpp1*^*asj/asj*^ animals were followed for reappearance of vascular and organ calcifications by weekly *in vivo* CT scans. Calcifications in both animals were eventually observed (cyan arrows). One animal developed calcifications in the heart on day 64, which progressed to the aorta, liver, kidney and spleen by day 79. The second animal developed renal calcifications on day 79. (**b**) Negative control experiment. To demonstrate that ENPP1-Fc enzyme activity is essential for therapeutic effect, and that weekly GK 1.5 administration does not alter the natural history disease, *enpp1*^*asj/asj*^ mice were dosed daily with 10 mg kg^−1^ inactive hENPP1-Fc, and weekly with GK 1.5 (blue diamonds, *n*=3). The average daily weights are plotted compared with *enpp1*^*asj/asj*^ mice dosed with vehicle—daily PBS_plus_ and weekly GK 1.5—(green triangles, *n*=9) and the error bars denote s.d.'s. All three *enpp1*^*asj/asj*^ mice dosed with inactive ENPP1-Fc experienced a drop in weight and mortality (deaths denoted by blue arrows) similar to *enpp1*^*asj/asj*^ mice dosed with vehicle (deaths denoted by red arrows), demonstrating that neither inactive ENPP1-Fc nor GK 1.5 extends survival.

To address questions regarding the reappearance of calcifications following the cessation of dosing, we enrolled two animals in a limited dosing trial in which *enpp1*^*asj/asj*^ animals were dosed with hENPP1-Fc starting on day 14 and ending on day 27. On day 28 and thereafter the animals were dosed with PBS_plus_ and the appearance of vascular calcifications were followed with weekly *in vivo* CT scans (Fig. 8a). Both animals were free of calcifications until day 64, when one animal developed calcifications in the heart, which were noted on day 79 to progress to the aorta, spleen, kidney, and liver. The second animal developed renal calcifications on day 79. Surprisingly both animals remained alive past day 84. The limited dosing study demonstrates that calcifications reappear following the cessation of dosing and that treatment in the 14–27 day window significantly extends survival in *enpp1*^*asj/asj*^ animals maintained on the acceleration diet *in utero*.

## Discussion

Diseases of ectopic tissue calcification range from ultra-rare diseases such as GACI to nearly ubiquitous maladies in the aging population such as hardening of the arteries. The genetic aetiology of human GACI suggests that the lethal arterial calcifications result from impairment of extracellular purinergic metabolism, either through loss of function mutations in ENPP1 or upstream reductions in nucleotide triphosphates metabolized by ENPP1 into extracellular PP_i_. Here we demonstrate that daily subcutaneous injections of ENPP1-Fc fusion protein eliminate the mortality, cardiac and arterial calcifications, and other sequela of disease in rodent models of GACI. ENPP1 is the enzyme responsible for the generation of extracellular PP_i_, and we demonstrate that ENPP1-Fc raises plasma pyrophosphate levels from the nearly undetectable levels present in *enpp1*^*asj/asj*^ mice to concentrations comparable to those seen in WT sibling pairs. Our findings suggest that ENPP1-Fc may be effective in other diseases of ectopic calcification in which plasma PP_i_ concentrations are decreased.

Extracellular PP_i_ is a potent inhibitor of mineralization. ENPP1 is the major producer, and tissue-nonspecific alkaline phosphatase (TNAP) is the primary degrader, of extracellular PP_i_ (Fig. 2a). In blood, [PP_i_] is ∼320 times lower than [P_i_], therefore small changes in [PP_i_] have large effects in the [P_i_]/[PP_i_] ratio, whereas similar magnitude changes in [P_i_] would not. If the [P_i_]/[PP_i_] ratio is the mechanism governing hydroxyapatite mineralization, as has been proposed, attention should be given to plasma [PP_i_] in patients suffering from disorders in ectopic mineralization.

The contribution of AMP generation and purinergic signalling to the suppression of vascular calcification is incompletely understood but has been addressed previous studies of *enpp1*^*asj/asj*^ mice using aortic allografts of WT into *enpp1*^*asj/asj*^ mice and vice versa^31^. These studies demonstrated that normal levels of extracellular pyrophosphate were sufficient to prevent vascular calcification over the entire surface of a transplanted *enpp1*^*asj/asj*^ aortic allograft. The suppression of calcification in aortic allografts was throughout the entire length of the allograft with no gradient of suppression of calcification observed. Because purinergic signalling is believed to be autocrine or paracrine in nature (and not systemic), the authors concluded that purinergic signalling was either not important for vascular calcification suppression or was not substantially altered by ENPP1 deficiency. Our observations that *enpp1*^*asj/asj*^ mice dosed with ENPP1-Fc are free of vascular calcifications and have normal plasma PP_i_ concentrations supports the notion that plasma PPi levels may be primarily responsible for suppression of vascular calcification in ENPP1 deficiency.

Reduced plasma PP_i_ levels are also present in vascular calcification associated with end stage renal disease (ESRD)^31^,^32^. One in ten adults suffer from chronic kidney disease (CKD), and it is estimated that 80% of patients with CKD on dialysis and 47–83% of patients with CKD not on dialysis possess vascular calcifications compromising their quality of life and endangering their health. Vascular calcifications associated with ESRD contributes to poor outcomes by increasing pulse pressure, causing or exacerbating hypertension, and inducing or intensifying myocardial infarctions and strokes. Most patients with ESRD do not die of renal failure, but from the cardiovascular complications of ESRD, and it is important to note that many very young patients with ESRD on dialysis possess coronary artery calcifications. The histologic subtype of vascular calcification associated with CKD is known as Mönckeburg's sclerosis, which is a form of vessel hardening in which calcium deposits are found in the muscular layers of the medial vascular wall. This form of calcification is histologically distinct from intimal or neo-intimal vascular wall calcification commonly observed in atherosclerosis but identical to the vascular calcifications observed in human GACI patients, and in the rodent models of the disease described herein. The similar pathophysiology of vascular calcification present in GACI and CKD suggests that these disorders may be treated by a common therapeutic.

To this point, the current medical strategies treating ectopic vascular calcification of CKD are typically ineffective and are directed at reducing hyperphosphatemia and minimizing serum calcium concentrations. Continuous intra-peritoneal infusions of exogenous PP_i_ substantially inhibits vascular calcifications in rodent models of ESRD, but the rapid hydrolysis of PP_i_ *in vivo* prevents translation of this therapy to the clinic^33^. Attempts treating vascular calcifications in rodent models of ESRD with non-hydrolyzable PP_i_ analogues (bisphosphonates) required doses above those known to inhibit bone formation, and the anticipated bone toxicity coupled with a renal mechanism of clearance discourages the use of bisphosphonates as therapeutic agents in CKD^34^. It appears that we are able to circumvent both the poor pharmacokinetics of PP_i_ and the bone toxicity of bisphosphonates by fusing the PP_i_ generating enzyme ENPP1 to the Fc domain of IgG1, and demonstrated that the biologic is effective in GACI. We suspect that it may also be effective in vascular calcification associated with CKD.

## Methods

### ENPP1-asj GACI mouse model

Animal care and maintenance were provided through Yale University Animal Resource Center at Yale University (New Haven). All procedures were approved by the Animal Care and Use Committee of Yale University and complied with the US National Institutes of Health guide for the care and use of laboratory animals. Heterozygous *enpp1*^*asj/+*^ (genotype C57BL/6J-Enpp1^asj^/GrsrJ, Jackson Laboratory stock number 012810) breeding pairs were maintained on the ‘acceleration diet' (TD.00442, Harlan Laboratories, Madison WI) throughout the entire experiment and food and water were delivered *ad libitum*. The animal colony was housed in pathogen free conditions. All experimental animals were maintained on the acceleration diet *in utero* through completion of the study. Litters were genotyped on day 8 and weaned at day 21. Following weaning, sibling pairs were sequentially divided into cohorts as described below and enrolled in experimental trials. Animals were consecutively enrolled in experimental trials without regard to gender, and the gender of the experimental animals was not recorded. On the basis of the trial dosing we estimated that statistically significant survival differences could be determined between dosed and undosed animals using a sample size of eight animals in each experimental group. We used identical breeding pairs throughout the study, and all experimental groups were enrolled sequentially beginning with the enpp1^wt^ and undosed *enpp1*^*asj/asj*^ cohorts and ending with the dosed *enpp1*^*asj/asj*^ cohort. Enrolment of the dosed and undosed *enpp1*^*asj/asj*^ cohorts spanned 4 months. Once the enrolment of an experimental group began both sexes of the appropriate genotype were consecutively enrolled in an experimental cohort with the exclusion of severely runted animals weighing <5.5 g at 14 days of life. Following weaning, all experimental animals were housed with littermates to allow for cooperative grooming and nesting. Experimentalists were not blinded during the study.

### ENPP1-Fc design

Human and mouse NPP1 (Human: NCBI accession NP_006199; Mouse: NCBI accession NP_03839) modified to express soluble, recombinant protein as described previously^26^ were fused to IgG_1_ by subcloning into pFUSE-h IgG_1_-Fc1 or pFUSE-m IgG_1_-Fc1 plasmids (InvivoGen, San Diego, CA), respectively. The final human protein sequence is listed in Supplementary Fig. 1.

### Protein production with shaking flasks

Stable transfections of the ENPP1-Fc were established in HEK293 cells under zeocin selection. Briefly, adherent HEK293 cells were adapted for suspension growth by progressive dilution of fetal bovine serum from 10 to 1% over 5–6 passages^35^. Zeocin selection (450 μg ml^−1^) was maintained throughout the adaptation process, and cultures were supplemented with 1% insulin-transferrin-selenium (Mediatech 25–800-CR), and cells were passaged when they reached confluence in 175 cm^2^ tissue culture flasks. Adapted cells were used to seed liquid culture growths in FreeStyle medium (Gibco, Waltham MA) in shaker flasks at 37°F and 5% CO_2_, agitated at 120 r.p.m. with high humidity. Cell density in liquid culure was maintained between 0.5 and 2.5 × 10^6^ cells per ml and zeocin selection was maintained in liquid culture at 450 μg ml^−1^ until the cells reached a volume of 4 l in liquid culture, and then reduced to 225 μg ml^−1^ thereafter. The culture was gradually expanded to 8 l and then maintained for another 12 days to accumulate extracellular protein. During the maintenance phase, cultures were supplemented with CD EfficientFeed C AGT (Gibco #A13275-05) to enhance protein production.

### Protein production with bioreactor

Cells were propagated in a 10 l bioreactor equipped with dissolved oxygen and pH control. Dissolved oxygen was kept at 40% air saturation by supplying the culture with mixture of air and oxygen not exceeding 3 l minute at an agitation rate of 80 RPM. pH was controlled at 7.4 by sparging CO2 when the pH was higher than 7.4. Culture growth was followed by measuring cell number, cell viability, glucose and lactate concentrations. Final yields for both methods of production were ∼5 mg of purified ENPP1-Fc per liter of culture.

### Protein purification

The liquid cultures were centrifuged at 4,300*g* for 15 min and the supernatants were filtered through a 0.2 μm membrane and concentrated via tangential low using a Pellicon3 0.11 m^2^ Ultracell 30 kD cassette (Millipore, Billerica MA). The concentrated supernatant was loaded onto a protein-A column, then washed using 50 mM Tris pH8, 150 mM NaCl_2_, 1 mM ZnCl_2_ and 1 mM CaCl_2_. The recombinant protein was eluted using Elution Buffer (150 mM Sodium Citrate; pH=4.3, 150 mM NaCl_2_, 1 mM ZnCl_2_ and 1 mM CaCl_2_), then immediately neutralized using a 1/10th volume of 1 M Tris pH 9.2, 150 mM NaCl_2_, 3 mM ZnCl_2_ and 3 mM CaCl_2_. Fractions containing enzymatic activity were pooled and dialysed against PBS_plus_ buffer (1 × PBS buffer pH 7.4, 11 uM ZnCl_2_, 20 uM CaCl_2_), then concentrated to ∼6 mg ml^−1^, distributed into small aliquots and stored at −80 °C. Resulting protein samples were then tested with Pierce LAL Chromogenic Endotoxin Quantitation Kit (cat. 88282) to verify that all were free of endotoxin.

### Enzymology

The steady state hydrolysis of ATP by human NPP1 was determined by high performance liquid chromatography (HPLC; refs 26, 36). Briefly, enzyme reactions were started by addition of 10 or 50 nM NPP1 to varying concentrations of ATP in the reaction buffer containing 20 mM Tris, pH 7.4, 150 mM NaCl, 4.5 KCl, 14 uM ZnCl2, 1 mM MgCl2 and 1 mM CaCl2. At various time points, 50 μl reaction solution was removed and quenched with an equal volume of 3 M formic acid. The quenched reaction solution was loaded on a C-18 (5 μm 250 × 4.6 mm) column (Higgins Analytical) equilibrated in 15 mM ammonium acetate (pH 6.0) solution and eluted with a 0–20% methanol gradient. Substrate and products were monitored by ultraviolet absorbance at 259 nm and quantified according to the integration of their correspondent peaks and standard curves.

### Size exclusion chromatography coupled with static laser light scattering

The light scattering data were collected using a Superose 6, 10/30, HR Size Exclusion Chromatography (SEC) column (GE Healthcare, Piscataway, NJ), connected to high performance liquid chromatography system (HPLC), Agilent 1200, (Agilent Technologies, Wilmington, DE) equipped with an autosampler. The elution from SEC was monitored by a photodiode array (PDA) UV/VIS detector, UV, (Agilent Technologies, Wilmington, DE), differential refractometer, RI, (OPTI-Lab rEx Wyatt Technology, Santa Barbara, CA), static and dynamic, multiangle laser light scattering (LS) detector (HELEOS II with QELS capability, Wyatt Technology, Santa Barbara, CA). The SEC-MALLS/UV/RI system was equilibrated in PBSplus buffer at the flow rate of 0.3 ml/min. Two software packages were used for data collection and analysis: the Chemstation software (Agilent Technologies, Wilmington, DE) controlled the HPLC operation and data collection from the multi-wavelength ultraviolet /VIS detector, while the ASTRA software (Wyatt Corp., Santa Barbara, CA) collected data from the refractive index detector, the light scattering detectors, and recorded the ultraviolet trace from the PDA detector. The weight average molecular weights, Mw, were determined across the entire elution profile in the intervals of 1 s from static light scattering (LS) measurement using ‘three detector approach'^27^,^28^,^29^.

### Vehicle

Mouse ENPP1-Fc (mENPP1-Fc) was formulated in vehicle such that the volume of vehicle delivered was 16 μl vehicle per gram of body weight. Vehicle consisted of americanBio 10X PBS (Stock# AB11072) diluted to 1 × with endotoxin free water and supplemented with 14 μM CaCl_2_ and 14 μM ZnCl_2_.

### Dosing

Animals in the proof of concept study (Figs 5, 6, 7) were dosed either with vehicle or with mouse ENPP1-Fc (mENPP1-Fc) formulated in vehicle. Mice were dosed with daily subcutaneous injections starting on day 14 at dose levels of 500 a.u. kg^−1^ mENPP1-Fc. Animals in the limited dosing study (Fig. 8a) were dosed with human ENPP1-Fc (hENPP1-Fc) formulated in vehicle beginning on the fourteenth day of life and ending on the twenty-seventh day of life. To tolerize the mice to human protein, the mice were dosed intraperitoneally with 75 μg of GK 1.5 (eBioscience, San Diego, CA) on the thirteenth day of life, and 50 μg of GK 1.5 on the twentieth day of life. Between days 28 and 83 the mice received vehicle. Inactive hENPP1-Fc protein (Fig. 8b) was generated by exposure to EDTA. The specific activity hENPP1-Fc following EDTA exposure was < 20 a.u. mg^−1^, or <50% of the activity of native protein.

### Clearance rate of ENPP1-Fc

Briefly, 15 male C57BI/6J mice aged 8 weeks or older were administered a single subcutaneous dose of 10 mg kg^−1^ of mENPP1-Fc. Blood was collected from tail veins into heparin-containing tubes before the injection and at different time points post injection. Mice were divided into three groups for blood collection: (1) 1 h, 8 h, 48 h, 96 h. (2). 3 h, 24 h, 45 h and 72 h. (3). 6 h and 32 h. Enpp1 activity in plasma were measured with pNP-TMP assay in the following buffer: 100 mM Tris·HCl (pH 9.0), 500 mM NaCl, 5 mM MgCl2, 0.05% (vol/vol) Triton X-100 and 4 mM pNp-TMP. Plasma activity units were converted into protein mass using an empirically determined conversion factor of 0.332 units per μg protein. To derive plasma concentrations and fraction of subcutaneous drug absorbed into plasma, the total blood volume of each mouse was estimated at 1.5 ml. Clearance rate constants were derived as detailed in the text.

### Definition of activity unit

Animals were dosed according to total activity of enzyme delivered rather than concentration of enzyme to account for varying activity among batches of enzyme used. One activity unit (1 a.u.) is defined as pM of pNP-TMP substrate hydrolyzed min^−1^ mg^−1^ enzyme. The activity assay was performed in a buffer consisting of 50 mM Tris pH9, 150 mM NaCl, 0.1 mM ZnCl_2_, 0.1 mM CaCl_2_, 0.1 mM MgCl_2_. The activity of acceptable protein preparations varied between 40 and 43 a.u. mg^−1^, and preparations with <40 a.u. mg^−1^ were discarded. A dose of 500 a.u. kg^−1^ corresponds to between 6 and 10 mg kg^−1^, depending on the specific activity of the protein preparation.

### Quantification of plasma PP_i_

Animals were terminally bled retro-orbitally using heparinized, micropipets, and the blood was immediately dispensed into heparin-treated eppendorf tubes and placed on wet ice. The samples were spun in a 4 °C pre-cooled microcentrifuge at 4,000 r.p.m. for 5 min, and plasma was collected and diluted in one volume of 50 mM Tris-Acetate pH=8.0. Plasma was then filtered through a 300 KDa membrane via ultracentrifugation (NanoSep 300 K, Pall Corp., Ann Arbour, MI) and frozen at −80 °C. Pyrophosphate was quantitated using standard three-step enzymatic assays using uridine 5' diphospho[^14^C]glucose to record the reaction product, uridine 5' diphospho[^14^C]gluconic acid^37^. Briefly, a reaction mixture (100 μl) containing 5 mM MgCl_2_, 90 mM KCL, 63 mM Tris-HCL (pH 7.6), 1 nmol NADP+, 2 nmol glucose 1,6-diphosphate, 400 pmol uridine 5'-diphosphoglucose, 0.02 μCi uridine 5' diphospho[^14^C]glucose, 0.25 units of uridine 5'-diphosphoglucose pyrophosphorylase, 0.25 units of phosphoglucose mutase, 0.5 units of glucose 6-phosphate dehydrogenase, and inorganic pyrophosphate (50–200 pmol) is incubated for 30 min at 37 °C. The reaction is terminated by the addition of 200 μl of 2% charcoal well suspended in water. The mixture is kept on ice, vortexed three times over 15 min and clarified by centrifugation for 5 min. An aliquote of 200 μl of supernatant is then counted in scintillation solution.

### *In vivo*^99m^PYP imaging

The bone imaging agent ^99m^Tc-pyrophosphate (Pharmalucence, Inc) was evaluated in cohorts of animals using a preclinical microSPECT/CT hybrid imaging system with dual 1 mm pinhole collimators (X-SPECT, Gamma Medica-Ideas)^38^. Each animal was injected intraperitoneally with 2–5 mCi of the radiolabelled tracer and imaged 1–1.5 h after injection. A CT scan (512 projections at 50 kVp, 800 uA and a magnification factor of 1.25) was acquired for anatomical co-localization with the SPECT image. The SPECT imaging was acquired with 180° per collimator head in a counter-clockwise rotation, 32 projections, 60 s per projection with an ROR of 7.0 cm, FOV of 8.95 cm and an energy window of 140 keV±20. CT images were reconstructed with the FLEX X-O CT software (Gamma Medica-Ideas) using a filtered back-projection algorithm. SPECT images were reconstructed using the FLEX SPECT software (5 iterations, 4 subsets) and subsequently fused with the CT images and analysed using the AMIRA software and offline in-house script. Data were corrected for decay and injected dose to achieve % injected dose.

### Quantification of ^99m^PYP uptake

For the ^99m^PYP murine scans, the animals were imaged two hours postinjection. The resulting SPECT scans were imported into NIH's ImageJ image processing software and regions of interest were drawn around each animal's head (target organ) and whole body. Per cent injected activity (PIA), often referred to as ‘per cent injected dose' was calculated by comparing the ratio of counts in the head to the counts in the whole body, and expressed as per cent injected dose to give a measure as of the affinity with which the radiotracer is taken up by the region of interest (head). The total counts in each scan were taken as the whole body measure of injected dose.

## Additional information

**How to cite this article:** Albright, R. A. *et al*. ENPP1-Fc prevents mortality and vascular calcifications in rodent model of generalized arterial calcification of infancy. *Nat. Commun.* 6:10006 doi: 10.1038/ncomms10006 (2015).

## Supplementary Material

Supplementary Figure and Note Supplementary Figure 1 and Supplementary Note 1

Supplementary Video 1 Clinical response of enpp1asj/asj mice to mENPP1-Fc treatment

Supplementary Video 2 Terminal CT scan of enpp1asj/asj mouse on acceleration diet dosed with vehicle

## Figures and Tables

**Table 1:** Cardiovascular pathology, natural history study.

	**WT**	**asj/asj**
Calcifications in heart (CT/histology)	0/0	37%/100%
Calcifications in aorta (CT/histology)	0/0	62%/100%
Calcifications in coronary arteries (histology)	0/0	100%
Myocardial infarction (histology)	0	70%

CT, computed tomography; WT, wild-type.

**Table 2:** Cardiovascular pathology, proof of concept study.

	**WT+Vehicle**	**asj/asj+vehicle**	**asj/asj+mENPP1-Fc**
Calcifications heart (CT/Histology)	0/0	55%/100%	0/0
Calcifications aorta (CT/Histology)	0/0	66%/100%	0/0
Calcifications in coronary arteries (CT/histology)	0/0	43%/100%	0/0
Myocardial infarction (Histology)	0/0	77%	0
Renal calcifications	60% (light)	100% (heavy)	100% (light)

CT, computed tomography; WT, wild-type.
