# Supplementary material for: ENPP1-Fc prevents mortality and vascular calcifications in rodent model of generalized arterial calcification of infancy
Source: Nat Commun. 2015 Dec 1;6:10006. doi: 10.1038/ncomms10006 (PMC4686714; doi:10.1038/ncomms10006)
Supplement: Supplementary Figure and Note — Supplementary Figure 1 and Supplementary Note 1 [file ncomms10006-s1.pdf]

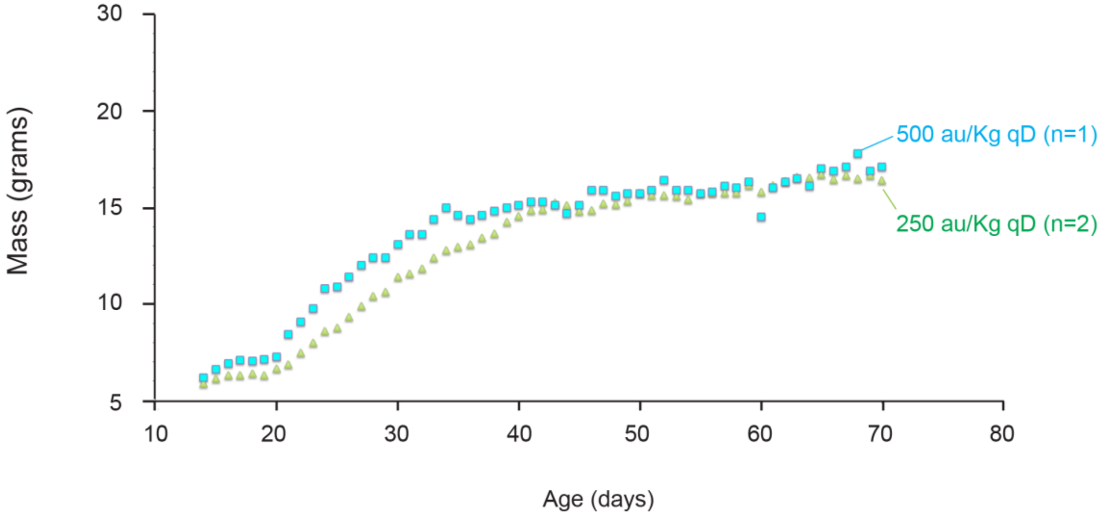

Supplementary Figure 1: Limited dose response study. The mean daily weights of *enpp1*$^{asj/asj}$ mice dosed with daily with either 250 au Kg^{-1} mENPP1-Fc (corresponding to ≈ 5 mg Kg^{-1}, green triangles, n=2), or 500 au Kg^{-1} mENPP1-Fc (corresponding to ≈ 10 mg Kg^{-1}, cyan squares, n=1) and weekly with Gk1.5 are plotted. Dosing and weighing commenced on day 14. Both dosing levels gave animals of comparable weights at about 45 days, but the higher dose gave an early and stronger response in animal weights, and was therefore chosen as the dose level for the definitive proof of concept study (Figure 5).

Supplementary Note 1:
Sequence Name: hENPP1-2-1-Fc

1	M	E	R	D	G	C	A	G	G	G	S	R	G	G	E	G	G	R	A	P	21
	R	E	G	P	A	G	N	G	R	D	R	G	R	S	H	A	A	E	A	P	41
	G	D	P	Q	A	A	A	S	L	L	A	P	M	D	V	G	E	E	P	L	61
	E	K	A	A	R	A	R	T	A	K	D	P	N	T	Y	K	I	I	S	L	81
	F	T	F	A	V	G	V	N	I	C	L	G	F	T	A	G	L	K	P	S	101
	C	A	K	E	V	K	S	C	K	G	R	C	F	E	R	T	F	G	N	C	121
	R	C	D	A	A	C	V	E	L	G	N	C	C	L	D	Y	Q	E	T	C	141
	I	E	P	E	H	I	W	T	C	N	K	F	R	C	G	E	K	R	L	T	161
	R	S	L	C	A	C	S	D	D	C	K	D	K	G	D	C	C	I	N	Y	181
	S	S	V	C	Q	G	E	K	S	W	V	E	E	P	C	E	S	I	N	E	201
	P	Q	C	P	A	G	F	E	T	P	P	T	L	L	F	S	L	D	G	F	221
	R	A	E	Y	L	H	T	W	G	G	L	L	P	V	I	S	K	L	K	K	241
	C	G	T	Y	T	K	N	M	R	P	V	Y	P	T	K	T	F	P	N	H	261
	Y	S	I	V	T	G	L	Y	P	E	S	H	G	I	I	D	N	K	M	Y	281
	D	P	K	M	N	A	S	F	S	L	K	S	K	E	K	F	N	P	E	W	301
	Y	K	G	E	P	I	W	V	T	A	K	Y	Q	G	L	K	S	G	T	F	321
	F	W	P	G	S	D	V	E	I	N	G	I	F	P	D	I	Y	K	M	Y	341
	N	G	S	V	P	F	E	E	R	I	L	A	V	L	Q	W	L	Q	L	P	361
	K	D	E	R	P	H	F	Y	T	L	Y	L	E	E	P	D	S	S	G	H	381
	S	Y	G	P	V	S	S	E	V	I	K	A	L	Q	R	V	D	G	M	V	401
	G	M	L	M	D	G	L	K	E	L	N	L	H	R	C	L	N	L	I	L	421
	I	S	D	H	G	M	E	Q	G	S	C	K	K	Y	I	Y	L	N	K	Y	441
	L	G	D	V	K	N	I	K	V	I	Y	G	P	A	A	R	L	R	P	S	461
	D	V	P	D	K	Y	Y	S	F	N	Y	E	G	I	A	R	N	L	S	C	481
	R	E	P	N	Q	H	F	K	P	Y	L	K	H	F	L	P	K	R	L	H	501
	F	A	K	S	D	R	I	E	P	L	T	F	Y	L	D	P	Q	W	Q	L	521
	A	L	N	P	S	E	R	K	Y	C	G	S	G	F	H	G	S	D	N	V	541
	F	S	N	M	Q	A	L	F	V	G	Y	G	P	G	F	K	H	G	I	E	561
	A	D	T	F	E	N	I	E	V	Y	N	L	M	C	D	L	L	N	L	T	581
	P	A	P	N	N	G	T	H	G	S	L	N	H	L	L	K	N	P	V	Y	601
	T	P	K	H	P	K	E	V	H	P	L	V	Q	C	P	F	T	R	N	P	621
	R	D	N	L	G	C	S	C	N	P	S	I	L	P	I	E	D	F	Q	T	641
	Q	F	N	L	T	V	A	E	E	K	I	I	K	H	E	T	L	P	Y	G	661
	R	P	R	V	L	Q	K	E	N	T	I	C	L	L	S	Q	H	Q	F	M	681
	S	G	Y	S	Q	D	I	L	M	P	L	W	T	S	Y	T	V	D	R	N	701
	D	S	F	S	T	E	D	F	S	N	C	L	Y	Q	D	F	R	I	P	L	721
	S	P	V	H	K	C	S	F	Y	K	N	N	T	K	V	S	Y	G	F	L	741
	S	P	P	Q	L	N	K	N	S	S	G	I	Y	S	E	A	L	L	T	T	761
	N	I	V	P	M	Y	Q	S	F	Q	V	I	W	R	Y	F	H	D	T	L	781
	L	R	K	Y	A	E	E	R	N	G	V	N	V	V	S	G	P	V	F	D	801
	F	D	Y	D	G	R	C	D	S	L	E	N	L	R	Q	K	R	R	V	I	821
	R	N	Q	E	I	L	I	P	T	H	F	F	I	V	L	T	S	C	K	D	841
	T	S	Q	T	P	L	H	C	E	N	L	D	T	L	A	F	I	L	P	H	861
	R	T	D	N	S	E	S	C	V	H	G	K	H	D	S	S	W	V	E	E	881
	L	L	M	L	H	R	A	R	I	T	D	V	E	H	I	T	G	L	S	F	901
	Y	Q	Q	R	K	E	P	V	S	D	I	L	K	L	K	T	H	L	P	T	921
	F	S	Q	E	D	L	I	N	D	K	T	H	T	C	P	P	C	P	A	P	941
	E	L	L	G	G	P	S	V	F	L	F	P	P	K	P	K	D	T	L	M	961
	I	S	R	T	P	E	V	T	C	V	V	V	D	V	S	H	E	D	P	E	981
	V	K	F	N	W	Y	V	D	G	V	E	V	H	N	A	K	T	K	P	R	1001
	E	E	Q	Y	N	S	T	Y	R	V	V	S	V	L	T	V	L	H	Q	D	1021
	W	L	N	G	K	E	Y	K	C	K	V	S	N	K	A	L	P	A	P	I	1041
	E	K	T	I	S	K	A	K	G	Q	P	R	E	P	Q	V	Y	T	L	P	1061
	P	S	R	E	E	M	T	K	N	Q	V	S	L	T	C	L	V	K	G	F	1081
	Y	P	S	D	I	A	V	E	W	E	S	N	G	Q	P	E	N	N	Y	K	1101

T T P P V L D S D G S F F L Y S K L T V 1121
D K S R W Q Q G N V F S C S V M H E A L 1141
H N H Y T Q K S L S L S P G K

Red-Cyan : Swapped residues with NPP2 residues 1-27 to give cleavage at the red-cyan transition.

Blue: hIgG1 (Fc)
Pink: NPP1 protein (beginning and end of extracellular region).
